# Drop‐Cast Hybrid Poly(styrene)‐b‐Poly(ethylene oxide) Metal Salt Films: Solvent Evaporation and Crystallinity‐Dependent Evolution of Film Morphology

**DOI:** 10.1002/smll.202406279

**Published:** 2024-10-13

**Authors:** Yanan Li, Nian Li, Suo Tu, Yamit Alon, Zerui Li, Marie Betker, Danzhong Sun, Alisher Kurmanbay, Wei Chen, Suzhe Liang, Shaowei Shi, Stephan V. Roth, Peter Müller‐Buschbaum

**Affiliations:** ^1^ Technical University of Munich TUM School of Natural Sciences Department of Physics Chair for Functional Materials James‐Franck‐Str. 1 85748 Garching Germany; ^2^ School of Physics University of Electronic Science and Technology of China Chengdu 610106 P. R. China; ^3^ Fibre and Polymer Technology KTH Royal Institute of Technology Teknikringen 56–58 Stockholm 11428 Sweden; ^4^ Deutsches Elektronen‐Synchrotron DESY Notkestrasse 85 22607 Hamburg Germany; ^5^ State Key Laboratory of Chemical Resource Engineering Beijing Advanced Innovation Center for Soft Matter Science and Engineering Beijing University of Chemical Technology Beijing 100029 P. R. China; ^6^ Shenzhen Key Laboratory of Ultraintense Laser and Advanced Material Technology Center for Intense Laser Application Technology and College of Engineering Physics Shenzhen Technology University Shenzhen 518118 P. R. China; ^7^ Eastern Institute for Advanced Study Eastern Institute of Technology Ningbo Zhejiang 315201 P. R. China; ^8^ University of Science and Technology of China Hefei Anhui 230026 P. R. China

**Keywords:** block copolymer, crystallinity, drop‐casting, metal salts, morphology

## Abstract

Morphology templates of solution–based diblock copolymer (DBC) films with loading metal salts are widely applied in photocatalysts, photovoltaics, and sensors due to their adjustable characteristics based on surface (de–)wetting and microphase separation. The present work investigates the morphologies of drop–cast hybrid films based on poly(styrene)–b–poly(ethylene oxide) (PS–b–PEO) and the metal salts titanium isopropoxide (TTIP) and zinc acetate dehydrate (ZAD) in comparison to the pure DBC. By utilizing scanning electron microscopy, grazing–incidence small– and wide–angle X‐ray scattering, and differential scanning calorimetry, we find that the resulting film morphologies depend not only on the presence of metal salts but also on solvent evaporation and crystalline formation. At 20 °C, additional TTIP and ZAD in the polymer template cause the morphology to change from packed globular structures to separated wormlike structures attributed to the changed polymer environment. Furthermore, additional tetrahydrofuran causes irregular structures at the precursor film part and the overlapped wormlike structures to transition into close–packed globular structures at the cap film parts of the pure DBC. In contrast, at 50 °C, the globular structures transit to fingerprint patterns due to the thermal behavior of the crystallizable PEO blocks, and the metal salt additives suppress crystalline structure formation in the PEO domains.

## Graphical abstract

The influences of solvent evaporation, loading metal salts, and concentration on drop–cast diblock copolymer templates adjusted by changing solution composition and temperature are investigated via X–ray scattering at subsequent positions throughout a film as a foundation for sensor fabrication. Heating plays an important role in the film consisting of crystallizable blocks, enlarging the parameter window for the diblock copolymer template.

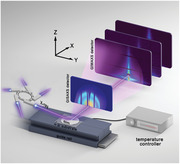

## 1 Introduction

Many device fabrication routes for photocatalysis, photovoltaics, and sensing utilize advanced liquid‐based deposition techniques, motivated by being cheap, simple, and working near room temperature.^[^ ^1^ ^]^ However, the theoretical prediction of thin film morphologies resulting from complicated non‐equilibrium processes, especially at interfaces, can be challenging. Thus, a deeper comprehension of the resulting interfacial‐film morphologies will be crucial for enhancing the performance of devices using such types of thin films. Given the broad interest in wet‐chemical processing, various solution‐based deposition methods for fabricating laboratory‐scale and industry‐scale films have been established,^[^ ^2^ ^]^ as, e.g., drop casting,^[^ ^3^ ^]^ spin coating,^[^ ^4^ ^]^ spray‐coating,^[^ ^5^ ^]^ ink‐jet type printing,^[^ ^6^ ^]^ and meniscus‐guided coating type printing.^[^ ^7^ ^]^ Spin coating is typically utilized for small‐scale film preparation. In contrast, spray‐coating and printing are used for large‐scale film preparation but require complicated setups and parameter adjustments. Among them, apparently, drop casting is the simplest and, depending on the drop size, is also relevant for most other deposition methods using inks. Despite their differences, a shared necessity, the effective regulation of the solution‐based film fabrication, exists for the available areas and morphology types. The mechanism varies depending on whether the polymer is in a dissolved or dispersed state.^[^ ^8^ ^]^ In solution‐based systems, film formation occurs as the solvent evaporates, while in polymeric dispersions, it relies on the coalescence of individual polymer spheres and the interpenetration of polymer chains. In addition, depending on the targeted resulting film, the spreading of the solution on the substrate^[^ ^9^ ^]^ and the adjustable liquid behavior during the drying process based on Marangoni flow,^[^ ^10^ ^]^ evaporation‐induced flow,^[^ ^11^ ^]^ and convective flow,^[^ ^12^ ^]^ need to be tailored to the needs. This is often accompanied by the formation of distinct film patterns from the solutions initially used. In particular, in the case of polymer films, various possible film morphologies depend not only on material concentrations and the use of additives but also on solvent parameters like the boiling point due to solvent evaporation during film fabrication.^[^ ^13^ ^]^ When targeting hybrid films composed of polymers and inorganic components, the complexity further increases. Typically, inorganic nanoparticles or precursor salts are added to the polymer solution in such a scenario. Nanoparticles and salt additives interact differently with the interfaces as the matrix polymer, which gives rise to enrichment or depletion processes driven by installed concentration gradients during the drying process. A valid simple approach to clarify the obtained morphologies is typically comparing the pure polymer template before and after loading the related nanoparticles or precursor salts prepared in different environments. Such morphology information plays an important role in improving the understanding of the properties of the final deposited hybrid films.^[^ ^14^ ^]^

Particular attention has been paid to diblock copolymers (DBCs) acting as templates. DBCs self‐assemble into various nanopatterns and, when being mixed with precursor salts, enable hybrid film synthesis via sol‐gel chemistry routes, facilitating the production of surfaces with multifunctional nanoscale features. However, the precursor salt additives influence the interfacial interactions in DBCs. This impacts on bulk morphologies as well as on film morphologies. For thin films, the interfacial interactions can result in pore/island formation and the (de‐)wetting from the substrate, in addition to the common microphase separation introducing the nanoscale microphase morphologies.^[^ ^15^ ^]^ Among the large number of precursor salts utilized for hybrid film formation, the metal ion‐containing precursors, such as, e.g., titania precursors^[^ ^16^ ^]^ and zinc precursors,^[^ ^17^ ^]^ have received particularly high interest. From the DBCs, amphiphilic polystyrene‐*block*‐poly(ethylene oxide) (PS‐*b*‐PEO) with a crystallizable PEO block has been used frequently in combination with these precursors.^[^ ^18^ ^]^ Despite the substantial body of work related to sol‐gel chemistry in combination with DBC templating, detailed comprehension of factors such as solvent evaporation differences and the specific crystalline behavior of PS‐*b*‐PEO segments with and without additives are still underestimated in comprehending the kinetically trapped morphologies during the film fabrication, e.g., via drop casting.^[^ ^19^ ^]^

The film morphologies with versatile structures are attributed to the (de‐)wetting of the DBC films on the surface in combination with the microphase separation being related to the length ratio between both blocks, the polymerization degree, and the temperature.^[^ ^20^ ^]^ Complications arise from the solvent evaporation, which can kinetically trap morphologies,^[^ ^21^ ^]^ the crystallization, which can overwrite the microphase separation or induce different morphologies,^[^ ^22^ ^]^ or interfacial energy effects from the interfaces to the substrate and to air.^[^ ^23^ ^]^ The resulting micro‐patterns are a consequence of the film‐free energy and kinetics,^[^ ^24^ ^]^ which govern the smaller‐scale textural details and drive diverse configurations in film morphology evolutions, offering control over complex pattern formations. For example, based on the morphology evolution of a pure PS‐*b*‐PEO (M_n_, PS = 12.3 kg mol^−1^ and M_n_, PEO = 14 kg mol^−1^) film from fingerprints into ripples by heating, Rice et al., determined the PEO melting temperature (T_m_) and the PS glass transition temperature (T_g_).^[^ ^25^ ^]^ However, the PS‐*b*‐PEO film morphology can probably also be attributed to the crystalline temperature (T_c_) of the semi‐crystalline PEO blocks, as noted by Rejek et al.,^[^ ^26^ ^]^ The crystalline PEO provides a robust framework to maintain the film integrity. In addition, it can also restrict the mobility and distribution of the additives (e.g., salts and nanoparticles) within the film, which finally will also influence a device's performance.^[^ ^27^ ^]^ Moreover, the temperature and the interaction between crystallizable PEO and additives also affect the film morphologies due to the changed environment of the PEO blocks.^[^ ^28^ ^]^ However, the temperature role depending on the crystalline PEO block in the PS‐*b*‐PEO film in combination with metal salt loading is still under‐explored.

In this work, we focus on the effect of the solvent evaporation rate and thermal behavior, being an easily controllable external parameter in the drop‐casting of films. We prepare films composed of pure PS‐*b*‐PEO and PS‐*b*‐PEO acting as a scaffold with metal salts at 20 and 50 °C via a sol‐gel process with drop casting due to its simplicity and importance to many other wet chemical processing methods. The polymer films are deposited from solutions of PS‐*b*‐PEO in the solvent mixtures with and without tetrahydrofuran (THF) to adjust the solvent evaporation rate. The polymer solutions are prepared based on the miscibility of dimethylformamide (DMF) with both THF and water, as well as the solubility of the polymer in this mixture. Therefore, our focus is primarily on the effect of solvent evaporation during film formation. The selected metal salts are titanium isopropoxide (TTIP)^[^ ^29^ ^]^ and zinc acetate dihydrate (ZAD),^[^ ^30^ ^]^ being prominent precursors of titanium dioxide and zinc oxide after calcination.^[^ ^31^ ^]^ We use mixtures of TTIP and ZAD to be incorporated into the DBC. Following the research of Rice et al.,^[^ ^25^ ^]^ further increasing the temperature can cause the film morphology to transition from fingerprint to ripple structures, which is not our target. With the comparison between 20 and 50 °C, we select very moderate temperatures to stay compatible with the ideas of low‐energy processing. At 20 °C, the self‐assembled microphase separation structures of PS‐*b*‐PEO mainly depend on the material concentration in the interplay with solvent evaporation. In contrast, the crystallization of the PEO blocks of the film treated at 50 °C acts as an additional driver in the system. It is possible that a combined effect could occur when increasing the temperature and adding volatile solvents, causing changes in the film morphology. Since heating improves the polymer chain mobility and speeds up solvent evaporation. However, in the present study, the temperature is higher than the melting point of the PEO block but below the melting point of the PS block, so that the core‐shell structures with flexible PEO chains remain stable. Therefore, we focus on the separate effects of temperature and solvent. We investigate the morphology evolution of the drop‐cast films with grazing‐incidence small‐angle X‐ray scattering (GISAXS) and scanning electron microscopy (SEM). As shown in Figure S1 (Supporting Information), the GISAXS measurements are done with a micro‐focused X‐ray beam to distinguish different film morphologies as a function of their distance to the triple‐phase contact line of the deposited drop. The thermal behavior of the polymers is characterized by differential scanning calorimetry (DSC). With grazing‐incidence wide‐angle X‐ray scattering (GIWAXS), information about the crystalline parts of the drop‐cast films is obtained.

## 2 Results and Discussion

PS‐*b*‐PEO and the metal salts TTIP and ZAD are dissolved in solvent mixtures to prepare polymer scaffolds and hybrid films, as shown in the Supporting Information. The solvent mixtures contain DMF, water, and THF. DMF is a good solvent for the solutes. The additional THF is utilized to adjust the boiling point of the mixture, whereas water facilitates the hydrolysis processes of TTIP and ZAD. Based on the calculated relative energy difference (RED)^[^ ^32^ ^]^ values of PS and PEO in the solvent mixtures (Table S1, Supporting Information), i.e., RED(PS) > RED(PEO), the resultant self‐assembly structures of PS‐*b*‐PEO manifest as micelles characterized by a PS core and a PEO shell. The formation of hybrid micelles with metal salts is driven by hydrogen bonding interactions, with TTIP and ZAD preferentially coordinating with the PEO shell. The film morphology adopted by the core–shell structural aggregates depends on a fine balance among the stretching of the polymer chains confined in the core, the interfacial tension between the core and solvent, and the interactions between the compressed corona chains. The surface morphology of a film can differ from the inner structures.^[^ ^15^, ^26^, ^33^ ^]^ Additionally, a typical feature of drop casting is the “coffee ring effect”, which results in increased solvent evaporation at the contact line than at the center of the droplet, so that the drop‐cast film can be split into two different parts denoted as precursor film being located in the vicinity of the contact line and cap film being the central part of the film (see Figure S2, Supporting Information). In the present study, the solvent evaporation rate is expected to strongly influence the BCP film topographies due to different evaporation rates as indicated in Figure S3 (Supporting Information).^[^ ^34^ ^]^

As well known, solution concentration, solvent mixture with different boiling points, and temperature emerge as the critical parameters for tuning the micellar structures and the final film morphologies. Thus, we choose two different concentrations (10 and 5 mg mL^−1^), two different solvent mixtures (DMF/water and DMF/water/THF), and two distinct temperatures (20 and 50 °C) and utilize the sol‐gel process combined with the drop‐casting method to prepare films on the pre‐cleaned silicon substrate with a hydrophilic surface. Figure S4a,b (Supporting Information) shows the topographies of the PS‐*b*‐PEO films from the 10 mg mL^−1^ PS‐*b*‐PEO dissolving in a mixture (DMF/water) solution prepared at 20 °C. Figure S4a (Supporting Information) represents the topography of the precursor film part, while Figure S4b (Supporting Information) illustrates the topography of the cap film part. The topographies evolve from the wormlike and hollow structures on the precursor film part to the wormlike and globular structures on the cap film part. The formed wormlike structures are attributed to the frozen‐in micellar structure of PS‐*b*‐PEO in the mixture of DMF and water as reported by Bhargava et al.,^[^ ^35^ ^]^ Thus, we assume that the appearance of globular structures on the cap film part can be attributed to the increasing concentration in this region. In this area, the formed wormlike structures transit from a lying‐down to a standing‐up state to a closed‐packed state for minimizing the film surface free energy.

To validate that the occurrence of the globular structures is caused by the increasing concentration due to solvent evaporation, we perform additional experiments at a lower concentration (Figure S5a,b, Supporting Information) and a lower boiling point (Figure S5c,d, Supporting Information). The drop casting is done with a solution of 5 mg mL^−1^ PS‐*b*‐PEO dissolving in a mixture (DMF/water) (Figure S5a,b, Supporting Information). As seen in Figure S5a (Supporting Information), the precursor film part primarily exhibits wormlike and hollow structures, with an increased occurrence of upright globular structures in the cap film part (Figure S5b, Supporting Information). Similarly, we observe that the structure size in the cap part is bigger than that of the precursor part when using the mixture solvent (DMF/water). Comparing Figures S4b and S5b (Supporting Information) indicates a noticeable reduction in the abundance of the globular structures (probably standing‐up wormlike structures) in Figure S5b (Supporting Information). Thus, it is reasonable that decreasing the cap film concentration produces the separated structures and hardly the transition from lying‐down to standing‐up state. Moreover, decreasing the boiling point of the solution also can change the concentration by accelerating the evaporation rate (de‐wetting process). As shown in Table S1 (Supporting Information), THF has a low boiling point and is miscible with DMF and water. In Figure S5c,d (Supporting Information), the effect of adding THF to the solution is shown for a solution of 5 mg mL^−1^ PS‐*b*‐PEO. Figure S5c (Supporting Information) shows worm‐like structures and large irregular areas, while Figure S5d (Supporting Information) showcases a combination of bent worm‐like, close‐packed hollow, and several globular structures. The fast evaporation causes the differences in the solute dispersion at the precursor film part and cap film part. Thus, the incorporation of THF has the potential to enhance the morphological diversity, fabricate close‐packed structures, and accentuate the distinctions between the precursor and cap parts in a film.

**Figure 1:**
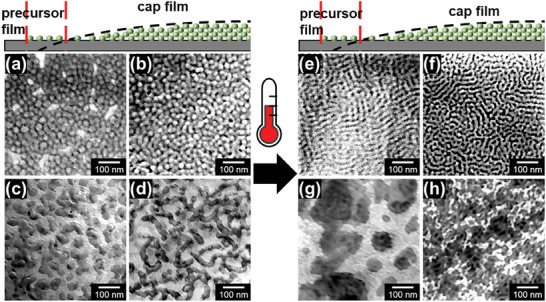
The surface morphology evolutions from the precursor film part to the cap film part of the polymer film (a,b,e,f) and the hybrid film (c,d,g,h) at 20 °C a–d) and 50 °C e–h). The films are prepared using the solution with solutes and the solvent mixture DMF/water/THF with 10 mg mL^−1^ PS‐*b*‐PEO. (a,b,e,f) Pure PS‐*b*‐PEO film; (c,d,g,h) hybrid film of PS‐*b*‐PEO and metal salts (TTIP and ZAD).

To confirm the above assumption and fabricate close‐packed globular structures on the cap film part, we prepare the polymer solution with 10 mg mL^−1^ PS‐*b*‐PEO in a mixture of DMF, water, and THF (see **Figure** **1**a,b). The morphology transits from vesicle structures with irregular areas (Figure 1a) to the desired close‐packed globular structures (Figure 1b). Therefore, we propose that increasing the concentration and accelerating the solvent evaporation rate drives the transition of wormlike structure from the initial lying‐down state to a standing‐up state, resulting in a denser globular structure arrangement. Additionally, the formed irregular areas in the precursor film part are attributed to the different humidities due to the solvent evaporation gradient in these local areas. Based on the above observations, the drop‐cast films prepared using a THF‐containing concentrated solution enable a diverse range of morphologies, specifically the close‐packed globular structures (standing‐up wormlike structures), which can be of interest for applications.

Notably, the drop‐cast film based on a solution with PS‐*b*‐PEO dissolving in the solvent mixture demonstrates promising potential application as a template for preparing metal oxide films with various morphologies after loading metal salts. To investigate this potential, we introduce TTIP and ZAD as metal salts into the PS‐*b*‐PEO template prepared at 20 °C, as shown in Figure 1c,d. The changes in the morphologies are attributed to the balancing of the interfacial energy between the two blocks (A‐*b*‐B) (an enthalpic contribution) and the entropic chain stretching energy of the individual blocks.^[^ ^36^ ^]^ The competition between these two opposing tendencies leads to nano‐scale phase separation,^[^ ^37^ ^]^ and the presence of salts in a BCP solution‐based film impacts the film morphology^[^ ^38^ ^]^ since additives like TTIP and ZAD have an effect on both energy contributions. When comparing the original polymer template (Figure 1a,b) with the hybrid films (Figure 1c,d), we see a change from packed globular to randomly dispersed worm‐like structures. The randomly dispersed worm‐like structures are attributed to the configuration of wormlike structures with electrostatic repulsive force, preventing them from approaching each other. Ren et al., illustrated the formation mechanism for the distances between the neighboring self‐assembled structures consisting of PS‐*b*‐PEO and acid due to electrostatic repulsive force.^[^ ^39^ ^]^

Fast evaporation is an efficient and economical way to prepare industrial‐scale films. Thus, besides decreasing the boiling point of the solution, increasing the temperature to accelerate the film preparation is also frequently utilized. However, increasing the temperature will not only affect the solvent evaporation but will also affect the polymer chain configuration, especially in the case of crystallizable polymers. To investigate such a scenario, the drop‐casting is done at an increased temperature of 50 °C (Figure 1e–h). The original globular structures observed at 20 °C (Figure 1a,b) undergo a morphological transition, resulting in the formation of fingerprint patterns in the center region (Figure 1e,f). This temperature‐induced change in morphology indicates the influence of temperature on the self‐assembly and organization of the film components during the drop‐casting process. Moreover, the addition of TTIP and ZAD leads to significant transformations in the film morphologies. In general, the homogeneity is reduced while the micro‐phase separation structure is still replicated to a certain extent (Figure 1g,h).

**Figure 2:**
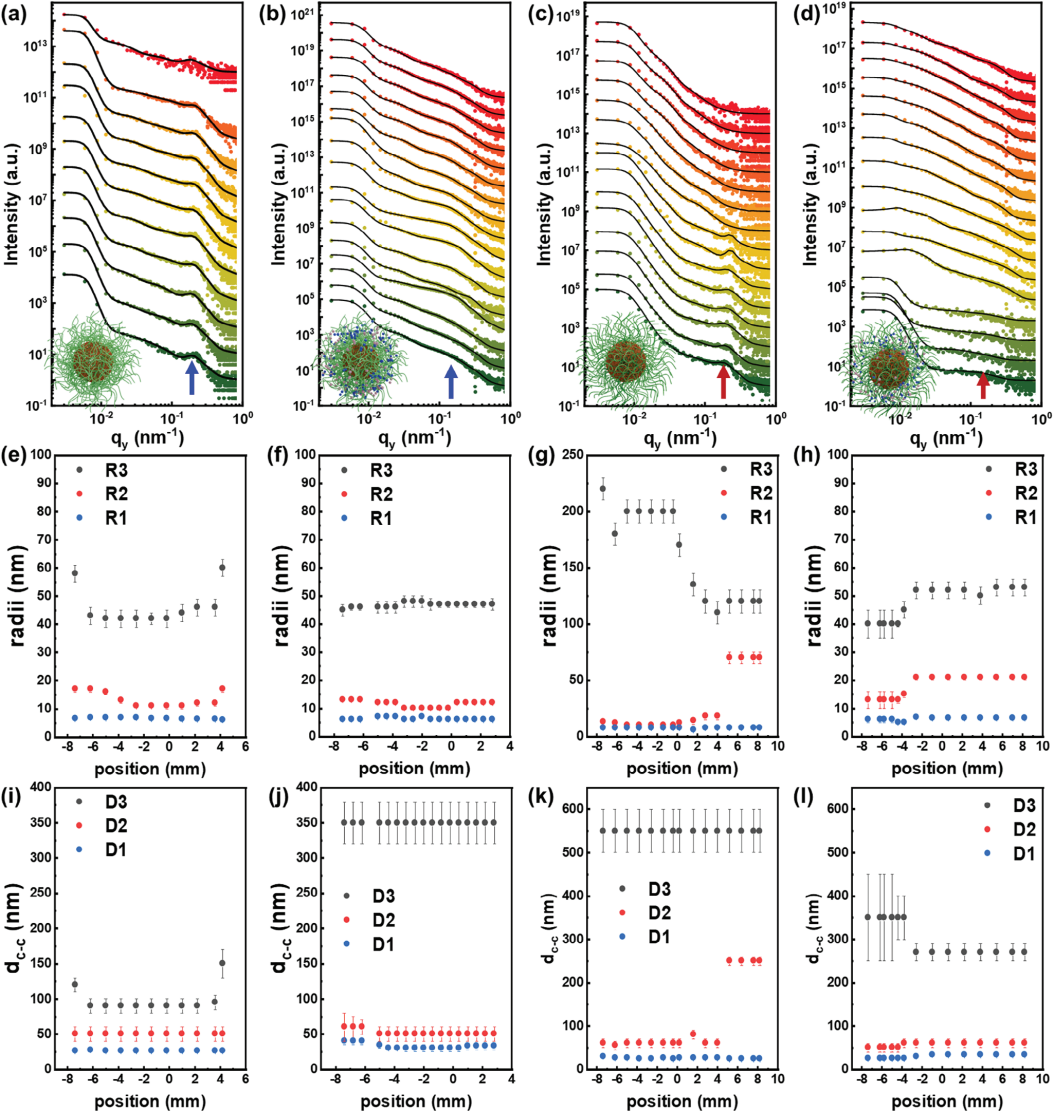
Selected horizontal line cuts (solid circles) of the 2D GISAXS data are shown together with corresponding fits (solid lines) from the scan through the drop‐cast films shown from green to red plots. For clarification, the data are shifted along the y‐axis. Films are prepared at a,b) 20 °C and c,d) 50 °C from (a,c) the pure DBC and (b,d) the hybrid DBC/metal salt‐containing solutions with 10 mg mL^−1^ PS‐*b*‐PEO in the mixtures. e–l) Characteristic structure parameters extracted from the modeling results are the center‐to‐center distances (D) and the radii (R). Black, red, and blue solid circle symbols denote object 1 (small structures), object 2 (medium structures), and object 3 (large structures) in the film morphology evolution as indicated. (e,f,i,j) the films prepared at 20 °C; (g,h,k,l) films prepared at 50 °C.

Since the film morphologies seen at the surface with SEM can differ from the inner film structures,^[^ ^21^, ^40^ ^]^ we use GISAXS in addition to the SEM measurements. To identify different morphologies between the precursor film parts and the cap film parts in the drop‐cast films, we scan the X‐ray beam laterally across the drop and record GISAXS data at different positions as indicated in Figure S6a (Supporting Information). This approach can reveal valuable information about the position‐dependent structure and its relationship to the film formation process, which is crucial for optimizing the fabrication process and improving the quality of the resulting films. To extract more detailed in‐plane structural information from these GISAXS data, we perform horizontal line cuts at the Yoneda peak position of 2D GISAXS data and fit the line cuts with a model based on three classes of cylindrically shaped objects (Figure S6b, Supporting Information) within the distorted wave Born approximation (DWBA), combining effective interface approximation (EIA) and local monodisperse approximations (LMA).^[^ ^41^ ^]^ The mapping presentation of the horizontal line cuts (Figure S7, Supporting Information) reveals the intensity evolution across different positions, clearly delineating the distinct regions identified earlier as the precursor and cap parts of the film. To see the quality of the fits, **Figure** **2**a–d shows selected horizontal line cuts (solid circles) of the films consisting of the pure DBC PS‐*b*‐PEO film (Figure 2a,c) and the hybrid film of PS‐*b*‐PEO loaded with metal salts (Figure 2b,d). In the horizontal line cut data of the PS‐*b*‐PEO films (Figure 2a,c), we observe a pronounced peak, which broadens for the hybrid films (Figure 2b,d) as indicated by the arrows. It results from ordered cylindrical objects of the polymer template, corresponding to the structures shown in SEM images in Figure 1a,b,e,f. The broadening of the peak in the hybrid films suggests a decrease in the degree of order compared to the PS‐*b*‐PEO films. Thus, the pronounced differences in the scattering curves are indicative of the different degrees of order as seen with SEM. Based on the information, we use three cylindrical structures to describe film morphology in GISAXS: The small structures of the self‐assembled micelles consist of PS core and PEO shell without metal salts. The middle structures represent the structures with metal salts or aggregates of small structures, and the large structures are aggregates, as illustrated in Figure S6b (Supporting Information). The corresponding fits are shown with solid lines (Figure 2a–d). The extracted values for the domain radii (R) and their corresponding center‐to‐center distances (D) are presented in Figure 2e–h and Figure 2i–l, respectively.

In the pure DBC film prepared at 20 °C (Figure 2e), R1 remains between 6.3 and 6.8 nm, while the value of R2 decreases from 17.0 to 11.0 nm and finally to 17.0 nm, and the value of R3 weakly increases from 43 to 46 nm, except for the two values at the film edges (58 and 60 nm). D1 remains constant at 26 nm, D2 stays at 50 nm, and D3 remains at 90 nm, with higher values at the edges (120 and 150 nm). (Figure 2i). This indicates a homogenous micelle template in the cap film, with structural changes in the precursor parts. In the hybrid DBC film prepared at 20 °C (Figure 2f), R1 slightly varies from 6.0 to 7.0 nm, R2 fluctuates from 13.0 to 10.0 nm, and R3 increases from 45 to 48 nm. D1 decreases from 40 to 30 nm and then increases to 33, while D2 decreases from 60 to 50 nm. D3 remains constant at 350 nm throughout the film. These observations suggest that the hybrid film behaves similarly to the original template with detailed differences.

In the pure DBC film prepared at 50 °C (Figure 2g), R1 remains stable at 7.5 nm, R2 increases from 13.0 to 70.0 nm, and R3 decreases from 220 to 120 nm. D1 stays at 26 nm except for the values of the film edges, D2 increases from 60 to 250 nm, and D3 remains constant at 550 nm. This indicates object packing from the precursor part to the cap part of the film. Compared to the film prepared at 20 °C, larger object sizes are observed at 50 °C due to reorganization. In the hybrid DBC film prepared at 50 °C (Figure 2h), R1 increases slightly from 6.0 to 6.5 nm, R2 increases from 13.0 to 21.0 nm, and R3 increases from 40 to 53 nm. Compared with the structure sizes of the pure polymer film prepared at a higher temperature, the large objects of the hybrid film are smaller, but the sizes of the small and medium objects are larger. This finding indicates that the metal salts reduce the aggregate formation and promote objects with more uniform dispersion. D1 increases from 25 to 33 nm, D2 increases from 50 to 60 nm, and D3 decreases from 350 to 270 nm. Thus, the film drying at 50 °C causes the large object to grow in comparison to the hybrid film prepared at 20 °C due to the aggregate formation induced by the fast solvent evaporation. The detailed values for the typical positions (Figure 2e–l) are provided in Table S2 (Supporting Information).

**Figure 3:**
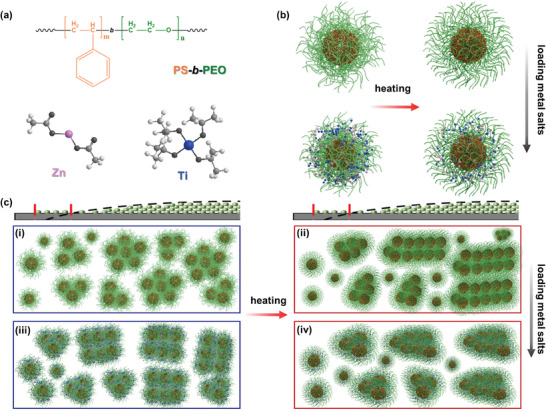
Schematic diagrams of a) PS‐*b*‐PEO diblock copolymer and metal salts including ZAD (pink), and TTIP (blue), b) nanostructures of the films via changing temperature and before and after loading metal salts, and c) the cross‐section view of the film inner‐structural evolutions from precursor film to cap film based on GISAXS measurements. These films contain (i, ii) PS‐*b*‐PEO or (iii, iv) PS‐*b*‐PEO with metal salts. Films of (i, iii) and (ii, iv) are prepared at 20 and 50 °C, respectively.

Based on these findings from the GISAXS data analysis, we make a schematic diagram of the inner film structure evolution from the precursor film part to the cap film part (**Figure** **3**). With the functions of solvent selectivity and evaporation, the DBCs undergo core–shell structures and a micro‐phase separation, and the objects evolve from loosely dispersed to tightly packed structures in transitioning from the precursor film to the cap film part. The additional metal salts (TTIP and ZAD) in the DBC film reduce the large‐object size, yet the sizes of other objects remain consistent throughout the film. Compared to the films at 20 and 50 °C, heating with fast evaporation produces larger aggregates for the DBC films, whereas heating does not cause the object size to grow significantly in the hybrid films. Thus, drop casting is indeed an alternative common method to obtain a morphology‐control hybrid film with homogeneous object sizes.

After identifying the role of solvent evaporation in guiding the film morphology, the crystalline behavior of the crystallizable PEO block is explored. For crystalline‐amorphous DBCs, the film morphology depends on the combination of crystallization and microphase separation.^[^ ^42^ ^]^ To provide information about the phase transitions of the template polymer PS‐*b*‐PEO, we perform the differential scanning calorimetry (DSC) measurements using the PS‐*b*‐PEO powder. Figure S8a–c and Table S3 (Supporting Information) show the glass transition temperature (*T_g_*), the melting temperature (*T_m_*) of PS and PEO blocks, and the crystalline temperature (*T_c_*) of PEO blocks. The films in the present work are prepared at 20 and 50 °C, which is close to the *T_c_* = 18 °C and *T_m_* = 52 °C of PEO. Thus, the film morphology changes at different temperatures are probably attributed to the behavior of semi‐crystalline PEO blocks.^[^ ^43^ ^]^ The temperatures of the drop‐cast films are both lower than *T_g_* (PS), thus the formed structures with PS core and PEO shell are always frozen‐in and supposed to no further transform. In addition, according to the three crystallization processes of polymers,^[^ ^43b^ ^]^ we assume that there are small crystal regions with defects in the PEO shell since 20 °C is close to *T_c_* of PEO blocks but much lower than *T_m_* (PEO); however, 50 °C is close to *T_m_* (PEO) so that the heat‐treated PEO chains can form large and well‐defined crystal domains in the film. Therefore, the films prepared at 20 and 50 °C show different morphologies as shown above. In addition, we obtain thermal information of the hybrid sample consisting of PS‐*b*‐PEO and metal salts by DSC measurements (Figure S8d–f and Table S3, Supporting Information). Comparing Figure S8a,d (Supporting Information), the number of crystalline peaks transits from two peaks to one peak on the cooling curve. This observation means that the introduction of metal salts in the sample has destroyed crystalline domains in the PEO shell.

**Figure 4:**
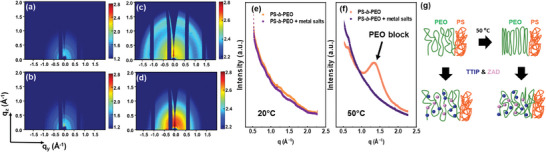
a–d) 2D GIWAXS data of the polymer film (a,c) and the hybrid film (b,d) at 20 °C (a,b) and 50 °C (c,d) for detecting the crystal structures. e,f) Radial cake cuts from the 2D GIWAXS data provide pseudo‐XRD information. g) Schematic illustration of the DBC chain transition by loading metal salts and heating.

To confirm the crystalline information of the polymer and hybrid film, we carry out GIWAXS measurements. **Figure** **4** displays the 2D GIWAXS data of the DBC films and hybrid films prepared at 20 and 50 °C. We find the characteristic crystallinity peaks of the PEO domain in the pure PS‐*b*‐PEO film prepared at 50 °C in a *q* range of (1.0–2.0) Å^−1^ (Figure 4c), while the effect of the metal salts on the PEO blocks inhibits or disrupts the crystallization process (Figure 4d) since the Bragg peaks disappear within the hybrid films. To obtain more details about the crystal structure, we perform the azimuthal integration of the 2D GIWAXS data, which provides pseudo‐X‐ray diffraction (pseudo‐XRD) data (Figure 4e,f). The films prepared at 20 °C show two weak bumps in a *q* range of (1.0–2.0) Å^−1^. However, the PS‐*b*‐PEO film prepared at 50 °C shows an obvious broad peak in the same range, which can be attributed to diffraction from (120), (112), and (111) planes of PEO crystallites.^[^ ^44^ ^]^ Such suppressed crystallization of one block has been known for a long. For example, in the context of lithium‐doped PS‐*b*‐PEO films, the addition of lithium salt can suppress the tendency of the PEO block to crystallize and the spacing increases with the additional salt. This behavior is attributed more to the conformation of the PEO chains than to the volume contribution of the added salts.^[^ ^45^ ^]^ Similar phenomena were extensively witnessed in previous investigations, which suggested that the spatial distribution of PEO‐selective components would reduce the chain length of crystallizable PEO and then inhibit the PEO crystallization due to the hydrogen‐bonding interactions.^[^ ^46^ ^]^ Therefore, the distribution of phase‐selective metal salts yields the transition from crystalline to amorphous PEO domains indicated in Figure 4g.

## 3 Conclusion

In summary, we study morphology in drop‐cast polymer versus hybrid films as they are used for templating metal salts in wet‐chemical deposited sol‐gel chemistry routes. By analyzing the precursor and cap film parts, we identify the morphological development across the potentially non‐homogenous film and identify the solvent evaporation rate and crystallization influencing it. Decreasing boiling temperature revealed that at 20 °C, the precursor film, after adding THF, appears to have irregular structures and a transition of cap films from wormlike structures to close‐packed bent‐wormlike and globular structures, underscoring the critical role of solvent evaporation in dictating the film morphology. The subsequent introduction of TTIP and ZAD as additives guides a further evolution from packed globular to separated wormlike structures. This shift is attributed to the alteration in interfacial energy, prompted by the extension of the PEO chains due to the presence of metal salts. Furthermore, due to the crystallizable PEO block, the study investigates the morphological changes at 20 and 50 °C, observing a progression from globular structures to fingerprint patterns. To illustrate the functions of the above factors on film inner structures, the modeling results show the three‐size objects remain stable except for the difference of large‐size objects on the polymer film prepared at 50 °C. The findings from this investigation highlight the significant influence of crystallinity on the morphological outcome of the PS‐*b*‐PEO template. The morphology difference between precursor and cap part when changing solvent evaporation and crystalline formation offers new insights into the phase behavior of PS‐*b*‐PEO and hybrid films, presenting a facile methodology for the creation of advanced semiconductor and sensor coatings with tailored properties.

## 4 Experimental Section

### Materials

The amphiphilic diblock copolymer template, poly(styrene‐*block*‐polyethylene oxide, (PS‐*b*‐PEO) was obtained from Polymer Source. Inc., Canada, and used as received. The number average molecular weights, M_n_, for the PS and PEO blocks, were 20.5 and 8.0 kg mol^−1^ respectively, with a polydispersity index of 1.02. The titania precursor, namely titanium (IV) isopropoxide (TTIP; 97%), was a transparent liquid with a relative density of 0.96 g mL^−1^; the zinc precursor, namely zinc acetate dehydrate (ZAD; 99.99% trace metal basis, a density of 1.84 g mL^−1^), was a white powder under room conditions. Except for the above two precursors, N, N‐dimethylformamide (DMF; analytical reagent grade 99.99%) and tetrahydrofuran (THF; 99.9%) were purchased from Sigma–Aldrich and used after the filtering.

### Characterization Methods

The surface morphologies of the pure polymer and hybrid films were characterized by high‐resolution field emission SEM (Zeiss Gemini NVision 40) at a working distance of 5 mm and an acceleration voltage of 3 kV. GISAXS and GIWAXS measurements were carried out on the P03 beamline of the PETRA III storage ring at DESY.^[^ ^47^ ^]^ The scattering signal was detected using a Pilatus 2 m (Dectris, pixel size 172 µm) detector and a LAMBDA 9 m (X‐Spectrum, pixel size 55 µm) detector (pixel size = 172 mm × 172 mm, pixel size = 55 mm × 55 mm).^[^ ^47^ ^]^ The following parameters were used: Wavelength was 1.044 Å, the sample‐to‐detector distance was 3550 mm for GISAXS measurements, the sample‐to‐detector distances were 261 and 243 mm for GIWAXS measurements, the X‐ray photon energy was 11.87 keV, and the incident angle was 0.3° and 0.12° for GISAXS and GIWAXS measurements, respectively. A Python program named Directly Programmable Data Analysis Kit (DPDAK) was used for calibration and data analysis.^[^ ^48^ ^]^ Horizontal line cuts of the 2D GISAXS data were performed at the Yoneda peak position. Details of the beamline are reported elsewhere.^[^ ^49^ ^]^ Analysis and integration of GIWAXS data was done using GIXSGUI software.^[^ ^50^ ^]^ Differential scanning calorimetry (DSC) was carried out using Mettler DSC25 in the temperature range of −80–150 °C at a heating rate of 10 °C min^−1^ under a nitrogen atmosphere.

## Conflict of Interest

The authors declare no conflict of interest.

## Supporting information



Supporting Information

## Data Availability

The data that support the findings of this study are available from the corresponding author upon reasonable request.
