# Supplementary material for: Drop‐Cast Hybrid Poly(styrene)‐b‐Poly(ethylene oxide) Metal Salt Films: Solvent Evaporation and Crystallinity‐Dependent Evolution of Film Morphology
Source: Small. 2024 Oct 13;20(51):2406279. doi: 10.1002/smll.202406279 (PMC11657052; doi:10.1002/smll.202406279)
Supplement: Supplementary file 1 — Supporting Information [file SMLL-20-2406279-s001.pdf]

NANO · MICRO
small

Supporting Information

for *Small*, DOI 10.1002/smll.202406279

Drop-Cast Hybrid Poly(styrene)-b-Poly(ethylene oxide) Metal Salt Films: Solvent Evaporation and Crystallinity-Dependent Evolution of Film Morphology

*Yanan Li, Nian Li, Suo Tu, Yamit Alon, Zerui Li, Marie Betker, Danzhong Sun, Alisher Kurmanbay, Wei Chen, Suzhe Liang, Shaowei Shi, Stephan V. Roth and Peter Müller-Buschbaum**

Supporting Information

Drop-Cast Hybrid Poly(styrene)-*b*-Poly(ethylene oxide) Metal Salt Films: Solvent Evaporation and Crystallinity-Dependent Evolution of Film Morphology

*Yanan Li, Nian Li, SuoTu, Yamit Alon, Zerui Li, Marie Betker, Danzhong Sun, Alisher Kurmanbay, Wei Chen, Suzhe Liang, Shaowei Shi, Stephan V. Roth, Peter Müller-Buschbaum**

Y. Li, S. Tu, Z. Li, S. Liang, P. Müller-Buschbaum
Technical University of Munich, TUM School of Natural Sciences, Department of Physics, Chair for Functional Materials, James-Franck-Str. 1, 85748 Garching, Germany

N. Li
School of Physics, University of Electronic Science and Technology of China, Chengdu 610106, China

Y. Alon, M. Betker, A. Kurmanbay, S. V. Roth
Fibre and Polymer Technology, KTH Royal Institute of Technology, Teknikringen 56-58, 11428 Stockholm

M. Betker, S. V. Roth
Deutsches Elektronen-Synchrotron DESY, Notkestrasse 85, 22607 Hamburg, Germany

D. Sun, S. Shi
State Key Laboratory of Chemical Resource Engineering, Beijing Advanced Innovation Center for Soft Matter Science and Engineering, Beijing University of Chemical Technology, Beijing 100029, China

W. Chen
Shenzhen Key Laboratory of Ultraintense Laser and Advanced Material Technology, Center for Intense Laser Application Technology, and College of Engineering Physics, Shenzhen Technology University, Shenzhen 518118, China

S. Liang
Eastern Institute for Advanced Study, Eastern Institute of Technology, Ningbo, Zhejiang 315201, P. R. China
University of Science and Technology of China, Hefei, Anhui 230026, P. R. China

Corresponding author E-mail: muellerb@ph.tum.de

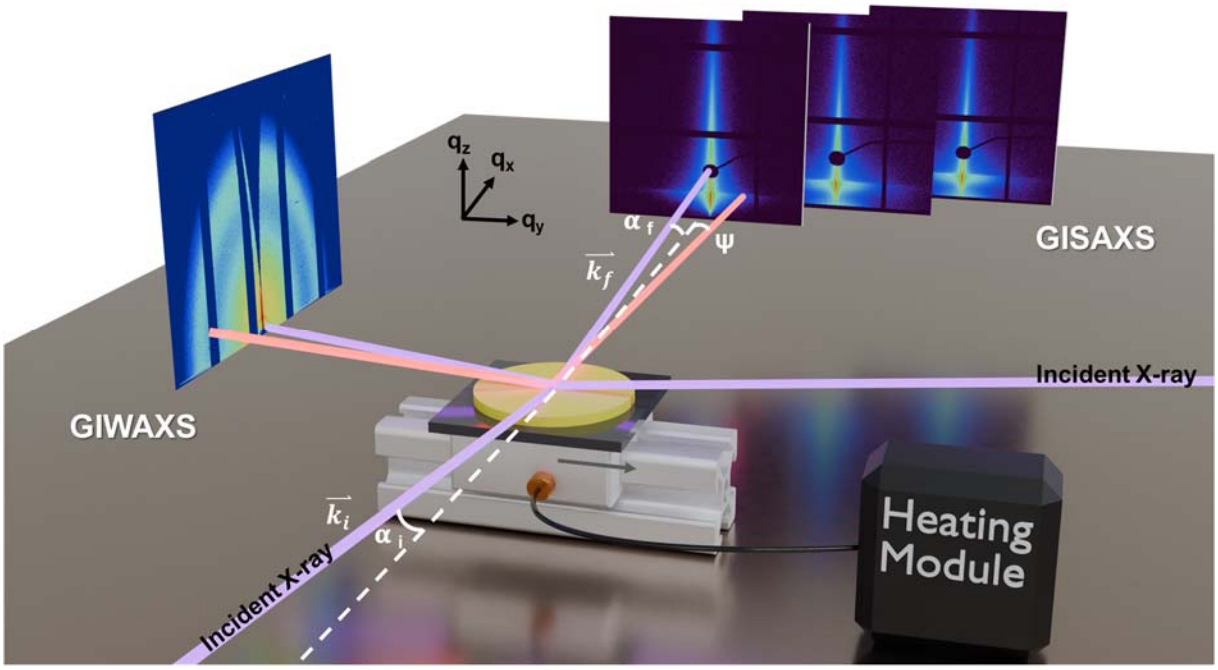

Figure S1. Schematic illustration of the setup used for studying film morphology at precursor and cap parts in case of drop-cast polymer and hybrid films prepared at different temperatures using GISAXS and GIWAXS scans. The studied film on the silicon substrate is moved via slide rails under the silicon substrate and heated with a heating module. The incident X-ray beam and reflected beam are shown in purple, while an exemplary scattered beam is shown in orange. The incoming X-ray beam (k_i) impinges at a small angle (α_i) with respect to the sample surface and the diffuse scattering is recorded using a 2D detector as a function of the exit angles (α_f and ψ).

Experimental Section

Sample preparation

We prepared two system solutions: Polymer solutions and a solution consisting of polymer and metal salts. The methods of DBC template loading with precursors were reported elsewhere.[1] For the preparation of the polymer solution, 15 mg polystyrene-*block*-poly(ethylene oxide) (PS-*b*-PEO) was dissolved in the mixture of 1500 uL DMF, 1200 uL THF and 240 uL DI water; 30 mg PS-*b*-PEO was dissolved in the mixture of 1500 uL DMF, 1200 uL THF and 240 uL DI water; 30 mg PS-*b*-PEO was dissolved in the mixture of 1500 uL DMF and 1500 uL DI water. We obtained a 5 mg mL^{-1} PS-*b*-PEO solution in a solvent mixture (DMF/THF/water = 1500/1200/240 uL), a 10 mg mL^{-1} PS-*b*-PEO solution in a solvent mixture (DMF/THF/water = 1500/1200/240 uL), and a 10 mg mL^{-1} of PS-*b*-PEO solution in a solvent mixture (DMF/ water = 1500/1500 uL). For the preparation of the solution containing polymer with metal salts (titanium isopropoxide, TTIP, and zinc acetate dihydrate, ZAD), 30 mg PS-*b*-PEO loading metal salts (60 mg TTIP and 120 mg ZAD) were dissolved in 1500 uL DMF. Then, 1200 uL of THF was added. Finally, 240 uL of DI water was slowly dropped into it. The whole process was carried out under magnetic stirring. Schematic views of the solution-based films shown in Figure S1 were prepared via drop casting of these solutions on pre-cleaned Si substrates at 20 °C and 50 °C, as shown in Figure S2. Water was utilized for the hydrolysis of the precursors (TTIP and ZAD); DMF and water are good solvents for PEO but poor solvents for PS, and THF is a good solvent for PS and PEO. THF and heating were utilized to adjust the solvent evaporation rate.

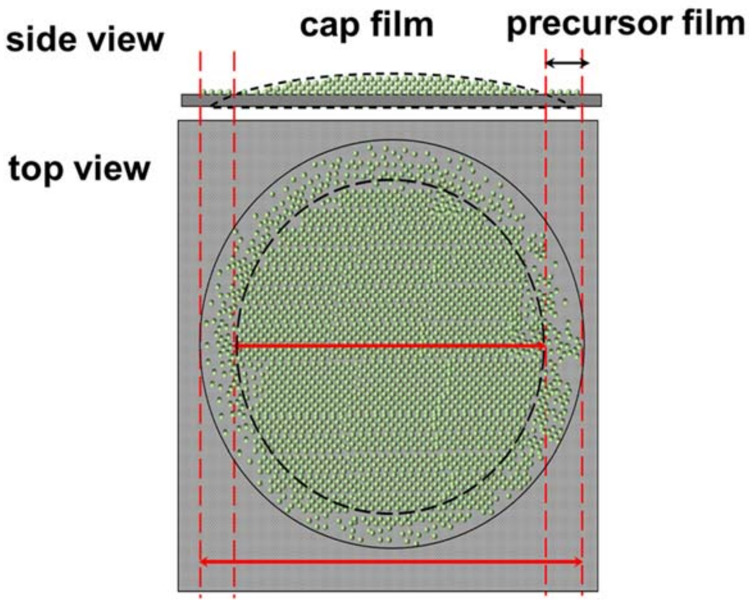

Figure S2. Schematic of the side view and top view of a drop-cast film and the two film parts denoted as precursor film part and cap film part.

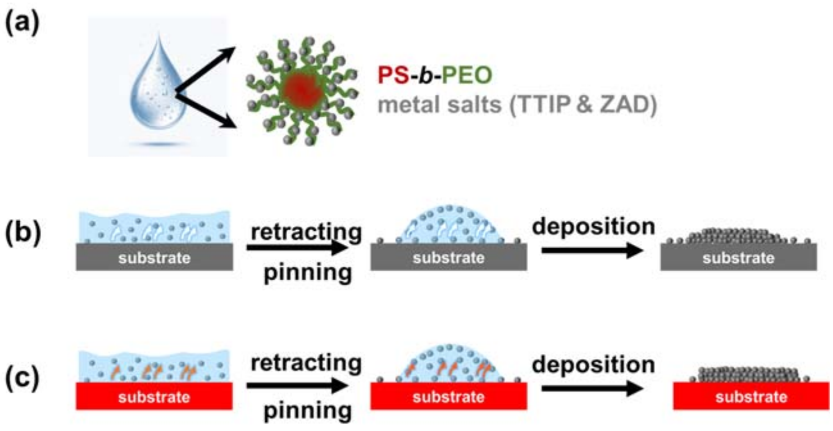

Figure S3. Schematic of (a) a solution of micelles composed of polystyrene-*block*-poly(ethylene oxide) (PS-*b*-PEO) and metal salts (titanium isopropoxide, TTIP, and zinc acetate dihydrate, ZAD). Sketch of the drop-cast film forming via solvent evaporation on the substrate at (b) 20 °C and (c) 50 °C.

Table S1. Parameters of polymers[2] and solvents[3].

materials \ parameters	boiling point (°C)	solubility parameters ($MPa^{1/2}$)								
		dispersion (D) δ_d	polar (P) δ_p	hydrogen bonding (H) δ_h	total solubility (T) δ_t	interaction radius (Ro)	Ra		Ra/Ro	
							PS	PEO	PS	PEO
PS	-----	21.3	5.8	4.3	22.5	12.7	-----		-----	
PEO	-----	21.5	10.9	13.1	27.4	15.9	-----		-----	
DMF	153.0	17.4	13.7	11.3	24.9	-----	13.1	8.8	1.0	0.6
Water	100.0	15.5	16.0	42.3	47.8	-----	41.0	32.0	3.2	2.0
THF	65-67	16.8	5.7	8.0	19.5	-----	9.7	11.9	0.8	0.7
D/W	126.5	16.5	14.9	26.8	34.8	-----	26.1	17.5	1.6	1.1
D/W/T	113.2	17.0	10.6	12.5	23.6	-----	12.8	9.0	1.0	0.6

The solubility of polymer solutions was analyzed by examining the Hansen solubility parameters (δ_d, δ_p, δ_h) in various solvents at room temperature. To determine the Hansen solubility parameters of PS and PEO, including δ_d, δ_p, δ_h, and Ro, the Gharagheizi[4] method was used. The value of Ra was calculated using the data obtained from the interaction of PS and PEO, which was selected as the solvent for film fabrication.

$$Ra^2 = 4(\delta_{d1} - \delta_{d2})^2+(\delta_{P1} - \delta_{P2})^2+(\delta_{h1} - \delta_{h2}) \qquad (1)$$

The solubility parameter was divided into three parts: δ_d, which represents the disperse bonding part, δ_p, which represents the polar bonding part, and δ_h, which represents the hydrogen bonding part. The solvent and polymer are represented by number 1 and number 2, respectively. The Hasen solubility parameters for the solvent mixture (DMF/water and DMF/water/THF) were calculated with the following Equation (2). The volume fraction of each solvent in a mixture was calculated using the following equation:

$$\delta_{DWT} = \delta_D (V_D/V_{tol}) + \delta_W (V_W/V_{tol}) + \delta_T (V_T/V_{tol})$$
$$V_{tol} = V_D + V_W + V_T. \qquad (2)$$

Hansen and Andersen[5] also defined the relative energy difference (RED) as the ratio RED=Ra/Ro[6]. A RED number of 0 indicates no energy difference. These numbers less than 1.0 indicate high affinity, while a value equal to or close to 1.0 represents a boundary condition and progressively lowers affinities.[7] Finally, according to the Ro of polymers, the relative energy difference (RED) value of PS and PEO in solvent and solvent mixtures was calculated in Table S1. The diblock copolymers PS-*b*-PEO dissolved in D/W and formed self-assembled structures with PS core and PEO shell since this solvent mixture is a good solvent for PEO but a bad solvent for PS, with RED (PS)=1.6 and RED (PEO)=1.1. PS-*b*-PEO dissolved in D/W/T and formed self-assembled structures with PS core and PEO shell since this solvent mixture is a good solvent for PEO but a bad solvent for PS, with RED (PS)=1.0 and RED (PEO)=0.6.

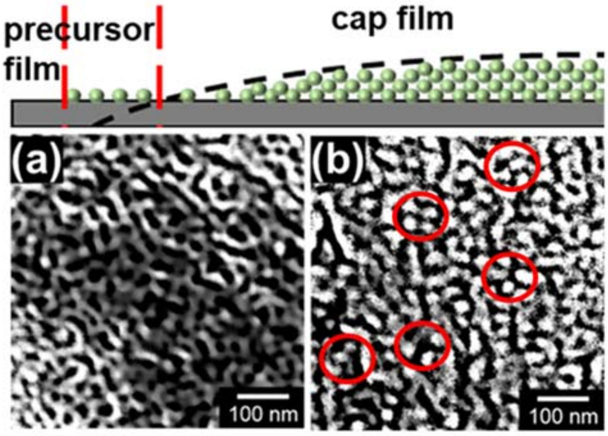

Figure S4. The top view of a drop-cast film and the two film parts are denoted as precursor film part (a) and cap film part (b). The film is prepared at 20 °C as seen in SEM images from wormlike and hollow to wormlike and globular structures seen in the red circles. These studies use 10 mg mL^{-1} of PS-*b*-PEO solution in a mixture of DMF/water shown in (a-b).

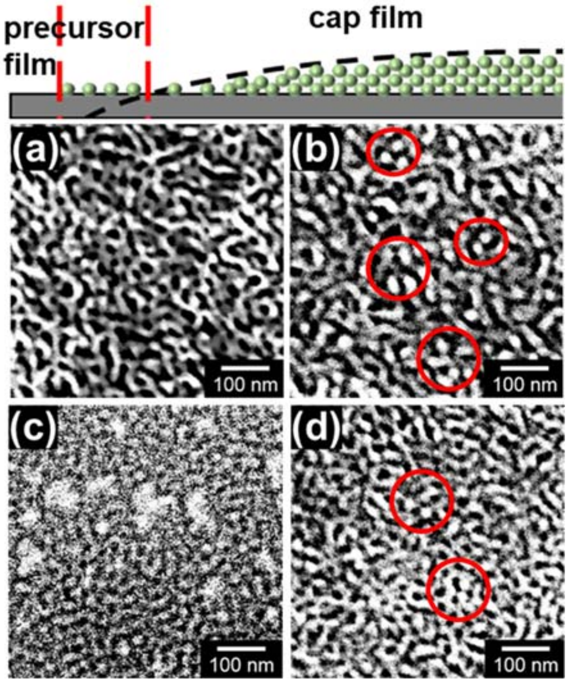

Figure S5. The surface morphology evolves from (a, c) the precursor film to (b, d) the cap film in the drop-cast film prepared at 20 °C as seen in SEM images from wormlike and hollow to wormlike and globular structures seen in the red circles. With the addition of THF, the structures change from (c) wormlike and irregular structures to (d) wormlike and hollow structures seen in the red circles. These studies use a 5 mg mL^{-1} PS-*b*-PEO solution in a mixture of DMF/water shown in (a-b) and a 5 mg mL^{-1} PS-*b*-PEO solution in a mixture of DMF/water/THF shown in (c-d).

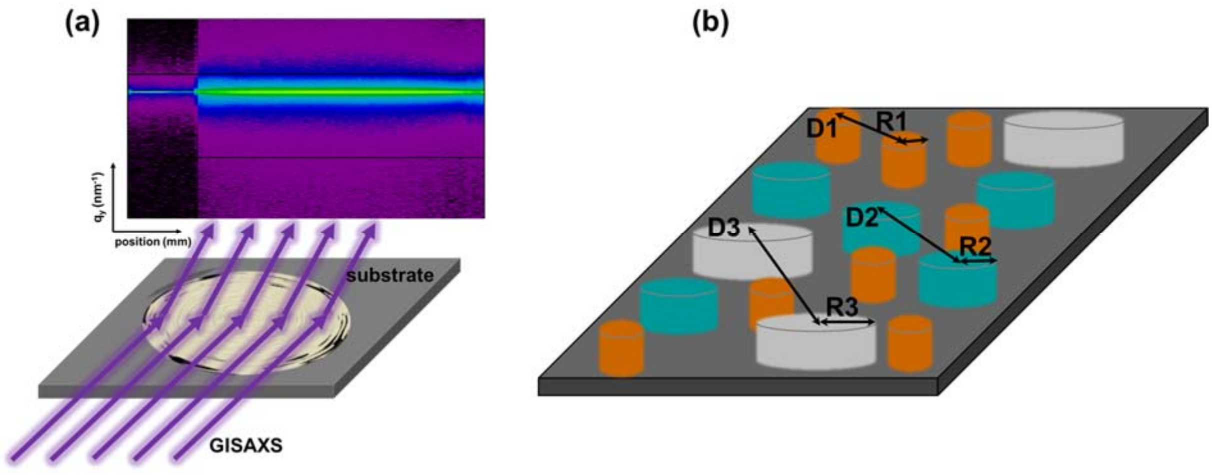

Figure S6. (a) Schematic diagram of GISAXS measurements of the drop-cast films performed at positions of a 5 uL drop by scanning the X-ray beam and resulting mapping of horizontal line cuts from the 2D GISAXS data. (b) Illustration of the model used to fit the GISAXS data assuming three classes of standing cylinders on the substrate. Structure factors (center-to-center distances, D) and form factors (radii, R) of the scattering objects are presented by arrows.

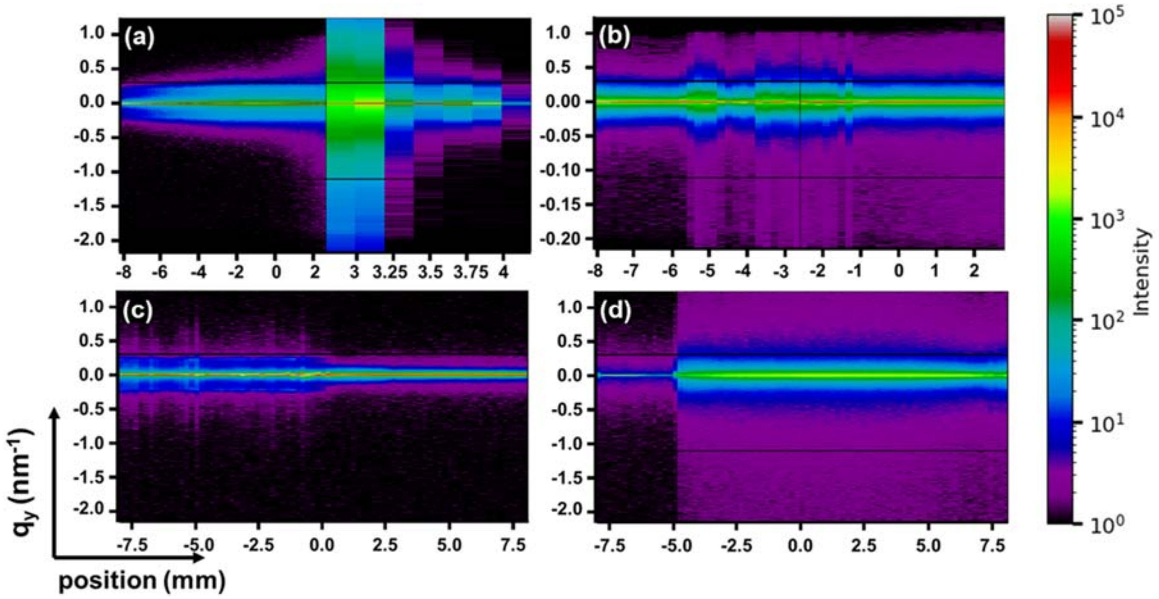

Figure S7. Mappings of horizontal line cuts extracted from the 2D GISAXS data plotted as a function of the position of the drop-cast film deposited at (a,c) 20 °C and (b,d) 50 °C in the case of (a,b) pure polymer and (c,d) hybrid films. All horizontal line cuts are integrated over the corresponding Yoneda region.

Table S2. Typical domain sizes of the films based on the modelling results in Figure 3e-l.

T (°C)	sample	position (mm)	domain radii			domain distances		
			R1 (nm)	R2 (nm)	R3 (nm)	D1 (nm)	D2 (nm)	D3 (nm)
20	PS-*b*-PEO	-7.4	6.5 ± 0.3	17.0 ± 1.0	58 ± 3	26 ± 1	50 ± 10	120 ± 10
		-6.2	6.8 ± 0.3	17.0 ± 1.0	43 ± 3	26 ± 1	50 ± 10	90 ± 10
		3.6	6.3 ± 0.3	11.0 ± 0.5	46 ± 3	26 ± 1	50 ± 10	95 ± 10
		4.2	6.0 ± 0.3	17.0 ± 1.0	60 ± 3	26 ± 1	50 ± 10	150 ± 10
	PS-*b*-PEO with metal salts	-7.4	6.0 ± 0.5	13.0 ± 0.5	45 ± 2	40 ± 5	60 ± 15	350 ± 30
		-2.0	7.0 ± 0.5	10.0 ± 0.5	48 ± 2	30 ± 5	50 ± 10	350 ± 30
		2.8	6.0 ± 0.5	12.0 ± 0.5	47 ± 2	33 ± 5	50 ± 10	350 ± 30
50	PS-*b*-PEO	-7.4	7.5 ± 0.5	13.0 ± 3.0	220 ± 10	29 ± 3	60 ± 10	550 ± 30
		-5.0	7.5 ± 0.5	10.0 ± 3.0	200 ± 10	26 ± 3	60 ± 10	550 ± 30
		5.2	7.5 ± 0.5	70.0 ± 5.0	120 ± 10	24 ± 3	250 ± 10	550 ± 30
	PS-*b*-PEO with metal salts	-7.4	6.0 ± 1.0	13.0 ± 3.0	40 ± 5	25 ± 4	50 ± 10	350 ± 100
		-1	6.5 ± 0.5	21.0 ± 1.0	52 ± 3	33 ± 1	60 ± 10	270 ± 20
		5.4	6.5 ± 0.5	21.0 ± 1.0	53 ± 3	33 ± 1	60 ± 10	270 ± 20

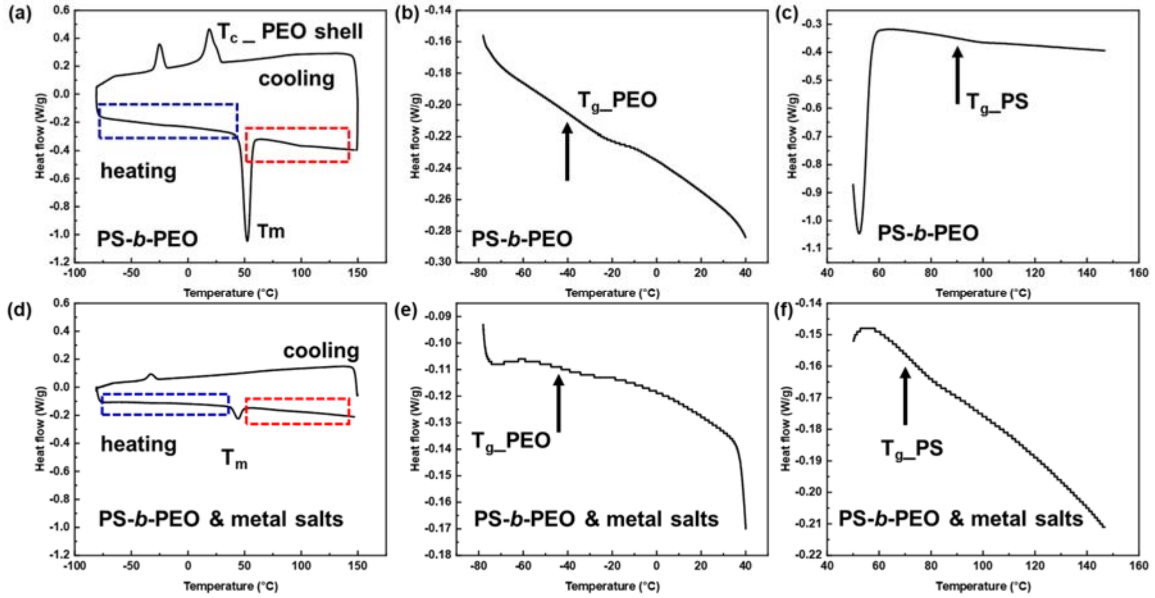

Figure S8. DSC curves (10 °C min^{-1}) of the powder containing PS-*b*-PEO (a-c) and the hybrid powder containing PS-*b*-PEO with metal salts (d-f) in the cooling run and the second heating run.

Table S3. Characteristic temperatures based on DSC measurements.

samples		T_c (ºC)	T_m (ºC)	T_g (ºC)
PS-*b*-PEO	PEO	-25, 18	52	-40
	PS	-----	-----	90
PS-*b*-PEO with metal salts	PEO	-33	44	-40
	PS	-----	-----	75

References

[1] L. Song, A. Abdelsamie, C. J. Schaffer, V. Körstgens, W. Wang, T. Wang, E. D. Indari, T. Fröschl, N. Hüsing, T. Haeberle, P. Lugli, S. Bernstorff, P. Müller-Buschbaum, *Adv. Funct. Mater.* **2016,** 26, 7084.
[2] a) A. F. Barton, *CRC Handbook of Solubility Parameters and Other Cohesion Parameters: Second Edition (2nd ed.)*. Routledge. **1991**; b) C. M. Hansen, *Hansen solubility parameters: a user's handbook*, CRC press, **2007**.
[3] A. F. Barton, *Chem. Rev.* **1975**, 75, 731.
[4] F. Gharagheizi, *J. Appl. Polym. Sci.* **2007**, 103, 31.
[5] C. M. Hansen, B. H. Andersen, *Am. Ind. Hyg. Assoc. J.* **1988**, 49, 301.
[6] A. Halperin, M. Tirrell, T. P. Lodge, *Macromolecules: Synthesis, Order and Advanced Properties* **1992**, 31.
[7] C. Özdemir, A. Güner, *Eur. Polym. J.* **2007**, 43, 3068.
